# Prognostic significance of the albumin-to-globulin ratio for upper tract urothelial carcinoma

**DOI:** 10.1186/s12894-020-00700-8

**Published:** 2020-08-28

**Authors:** Shota Omura, Satoru Taguchi, Shogo Miyagawa, Ryuki Matsumoto, Mio Samejima, Naoki Ninomiya, Kazuki Masuda, Yu Nakamura, Tsuyoshi Yamaguchi, Manami Kinjo, Mitsuhiro Tambo, Takatsugu Okegawa, Eiji Higashihara, Hiroshi Fukuhara

**Affiliations:** 1 Department of Urology, Kyorin University School of Medicine, 6-20-2 Shinkawa, Mitaka, Tokyo, 181-8611 Japan; 2 Department of ADPKD Research, Kyorin University School of Medicine, 6-20-2 Shinkawa, Mitaka, Tokyo, 181-8611 Japan

**Keywords:** AGR, Albumin-to-globulin ratio, Biomarker, Radical nephroureterectomy, REMARK, Upper tract urothelial carcinoma

## Abstract

**Background:**

Although the albumin-to-globulin ratio (AGR) is a promising biomarker for various malignancies, few studies have investigated its prognostic significance for upper tract urothelial carcinoma (UTUC).

**Methods:**

This retrospective study conformed to the REporting recommendations for tumour MARKer prognostic studies (REMARK) guideline. We reviewed 179 patients with UTUC who underwent radical nephroureterectomy at our institution between 2008 and 2018. Associations of preoperative clinicopathological factors, including the AGR, with cancer-specific survival (CSS) and overall survival (OS) were assessed. The Cox proportional hazards model was used for univariate and multivariable analyses. AGR was dichotomized as < 1.25 and ≥ 1.25, according to the most discriminatory cutoff determined from the receiver operating characteristic curve analysis.

**Results:**

During a median follow-up of 34 months after surgery, 37 patients died from UTUC and 13 died of other causes. The preoperative AGR significantly correlated with pathological T stage, pathological N stage, and adjuvant chemotherapy. Multivariate analyses demonstrated that a decreased (< 1.25) preoperative AGR was an independent poor prognostic factor for both CSS (hazard ratio [HR] = 2.81, *P* <  0.01) and OS (HR = 2.09, *P* <  0.05).

**Conclusions:**

Preoperative AGR < 1.25 might serve as a useful prognostic marker for patients with UTUC undergoing radical nephroureterectomy.

## Background

Upper tract urothelial carcinoma (UTUC) is a relatively rare malignancy that accounts for 5–10% of urothelial carcinomas and generally has a poor prognosis [1, 2]. Radical nephroureterectomy with bladder cuff excision is the gold standard treatment for nonmetastatic UTUC [3], whereas up to 19% of patients with UTUC have metastasis upon initial presentation [4].

Clinicopathological factors [5–15] including laboratory markers [11–15] serve as prognostic markers for UTUC. In contrast, the significance of the albumin-to-globulin ratio (AGR), which serves as a useful biomarker for various malignancies [16–26], has not been fully investigated in UTUC [23–26]. Therefore, the present study assessed the significance of the association of preoperative AGR on oncological outcomes of patients with UTUC undergoing radical nephroureterectomy.

## Methods

This retrospective study conformed to the REporting recommendations for tumour MARKer prognostic studies (REMARK) guideline [27] (Supplementary Table 1 shows the REMARK checklist of the present study). This study was approved by the internal institutional review board of Kyorin University School of Medicine (approval number: 1154).

### Patients

We retrospectively reviewed 185 consecutive patients who underwent radical nephroureterectomy with curative intent at Kyorin University Hospital between 2008 and 2018. We excluded six patients because of pathological diagnoses of urothelial dysplasia (*n* = 3), renal cell carcinoma (*n* = 2), and squamous cell carcinoma (*n* = 1), which left 179 available for analysis.

### Preoperative AGR

Routine preoperative blood tests including serum total protein and albumin levels (g/dl) were performed within 1 month before surgery. The AGR was calculated using the following formula: AGR = [albumin / (total protein – albumin)]. No patient had active infectious disorders during the blood tests.

### Endpoints and follow-up

We assessed the associations of preoperative clinicopathological factors, including the AGR, with cancer-specific survival (CSS) and overall survival (OS). The follow-up period started on the day of surgery. Follow-up information was obtained as of October 2018.

### Statistical analysis

Receiver operating characteristic (ROC) curve analysis was used to determine the optimal cutoff value of the AGR. Sensitivity, specificity and area under the curve (AUC) were calculated using a 2 × 2 contingency table for different cutoff values of the AGR. The optimal cutoff value of the AGR was determined by maximization of the Youden’s index [Sensitivity − (1 − Specificity)]. Relations of the AGR to other variables were evaluated using the χ^2^ test or Spearman’s rank correlation coefficient. Survival curves were generated using the Kaplan–Meier method and compared using log-rank tests. The Cox proportional hazard regression model was used for univariate and multivariate analyses. All statistical analyses were performed using JMP Pro version 14.0.0 (SAS Institute, Cary, NC, USA). *P* <  0.05 was considered to indicate a significant difference.

## Results

**Fig. 1:**
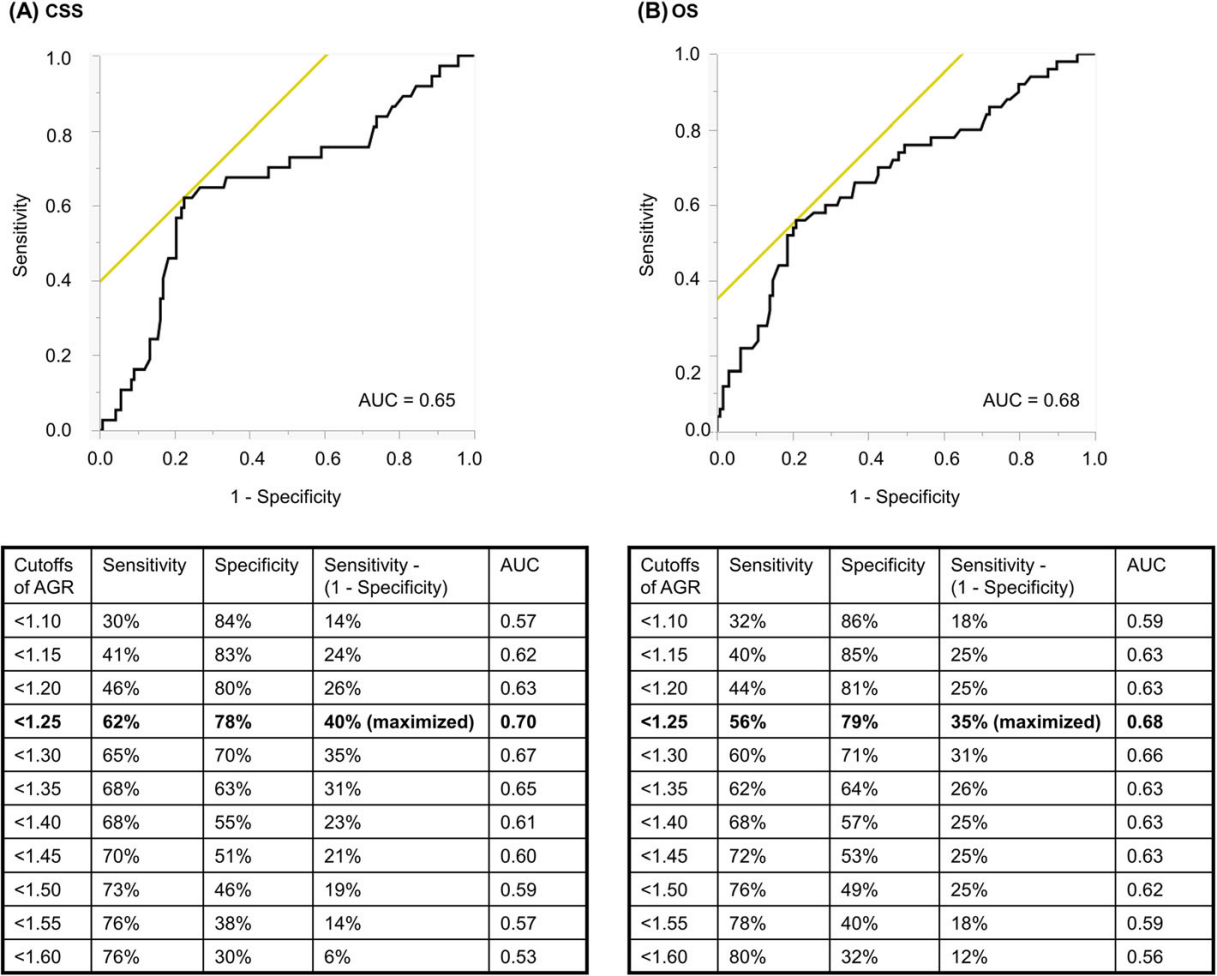
ROC curve analyses for (**a**) CSS and (**b**) OS. The optimal cutoff value of AGR was estimated as < 1.25 by maximizing the Youden’s index [Sensitivity − (1 − Specificity)] for both endpoints of CSS and OS. Abbreviations: AGR = albumin-to-globulin ratio; CSS = cancer-specific survival; OS = overall survival; ROC = receiver operating characteristic

Patients’ baseline characteristics are summarized in Table 1. The median follow-up was 34 months (interquartile range [IQR], 17–63) months. Thirty-seven patients died from UTUC and 13 died of other causes. ROC curve analysis identified 1.25 as the most discriminatory cutoff value of AGR by maximization of the Youden’s index [Sensitivity − (1 − Specificity)] for both endpoints of CSS and OS (Fig. 1).

**Table 1:** Patient characteristics (*n* = 179)

Parameter	Value
Age at surgery, years, median (IQR)	75 (66–79)
Sex, no. (%):	
Male	132 (73.7)
Female	47 (26.3)
Surgical technique, no. (%):	
Open	16 (8.9)
Laparoscopic	163 (91.1)
Tumor location, no. (%):	
Renal pelvis	96 (53.6)
Ureter	81 (45.3)
Both	2 (1.1)
Tumor grade, no. (%):	
G1	10 (5.6)
G2	94 (52.5)
G3	75 (41.9)
Pathological T stage, no. (%):	
Ta/1/is	91 (50.8)
T2	13 (7.3)
T3	70 (39.1)
T4	5 (2.8)
Pathological N stage, no. (%):	
N0/x	157 (87.7)
N1	16 (8.9)
N2	6 (3.4)
N3	0 (0)
Adjuvant chemotherapy, no. (%):	
Yes	39 (21.8)
No	140 (78.2)
Bladder cancer status, no. (%):	
No	145 (81.0)
Previous	19 (10.6)
Synchronous	15 (8.4)
AGR, median (IQR)	1.41 (1.18–1.63)
Follow-up duration, months, median (IQR)	35 (17–63)

Abbreviations: *AGR* albumin-to-globulin ratio, *IQR* interquartile range

χ^2^ test revealed pathological T stage (≥T3, *P* <  0.01), pathological N stage (N1–3, *P* <  0.01), and adjuvant chemotherapy (yes, *P* <  0.05) to be significantly associated with AGR < 1.25, while the other variables (sex, surgical technique, tumor location, tumor grade and previous or synchronous bladder cancer) were not. Spearman’s rank correlation coefficient showed a weakly significant negative correlation between age at surgery and the AGR (ρ = − 0.25, *P* <  0.01).

**Fig. 2:**
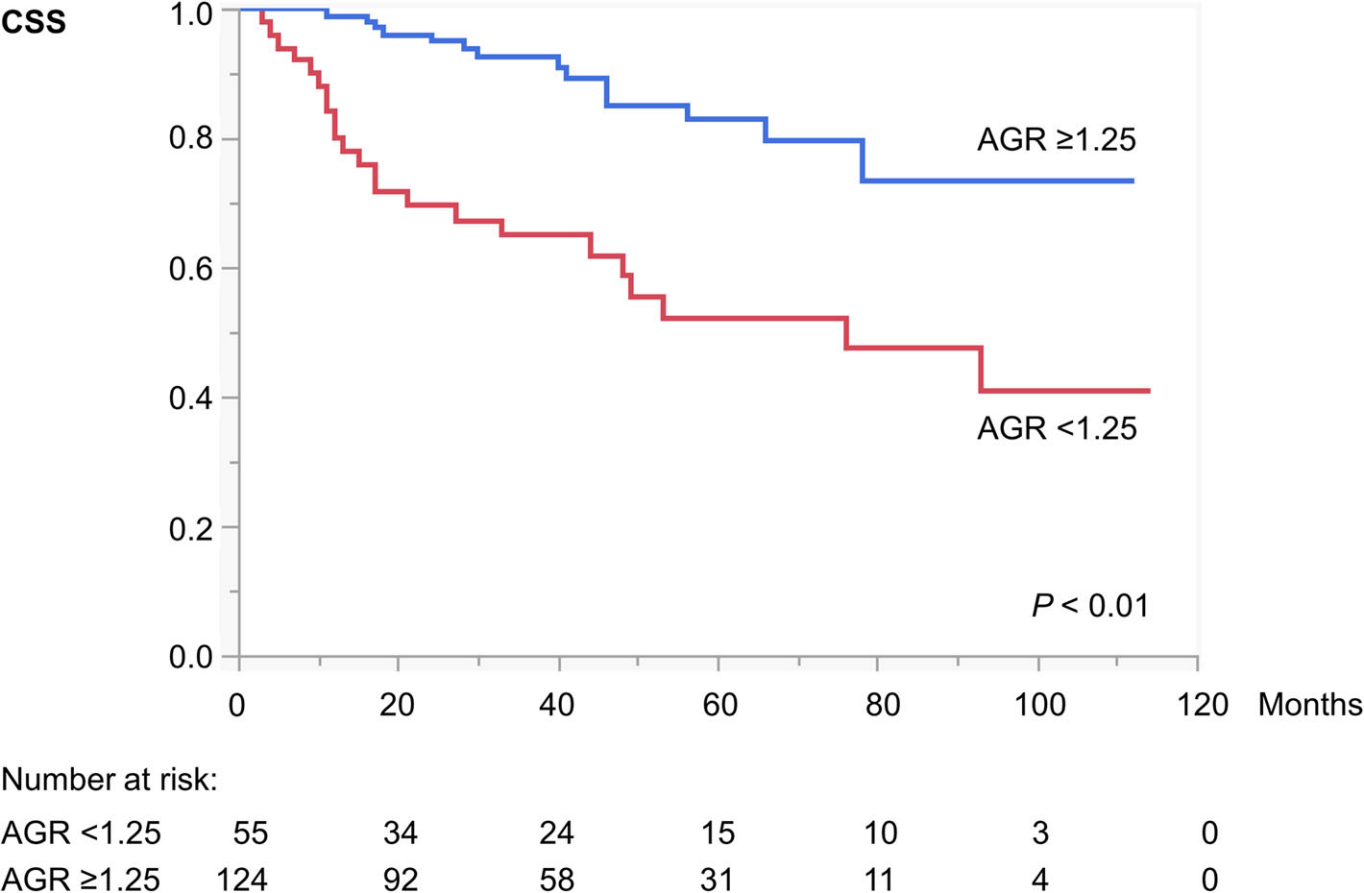
Kaplan–Meier curves depicting CSS in patients with AGR < 1.25 versus ≥1.25 (*P* < 0.01, log-rank test). Abbreviations: AGR = albumin-to-globulin ratio; CSS = cancer-specific survival

**Fig. 3:**
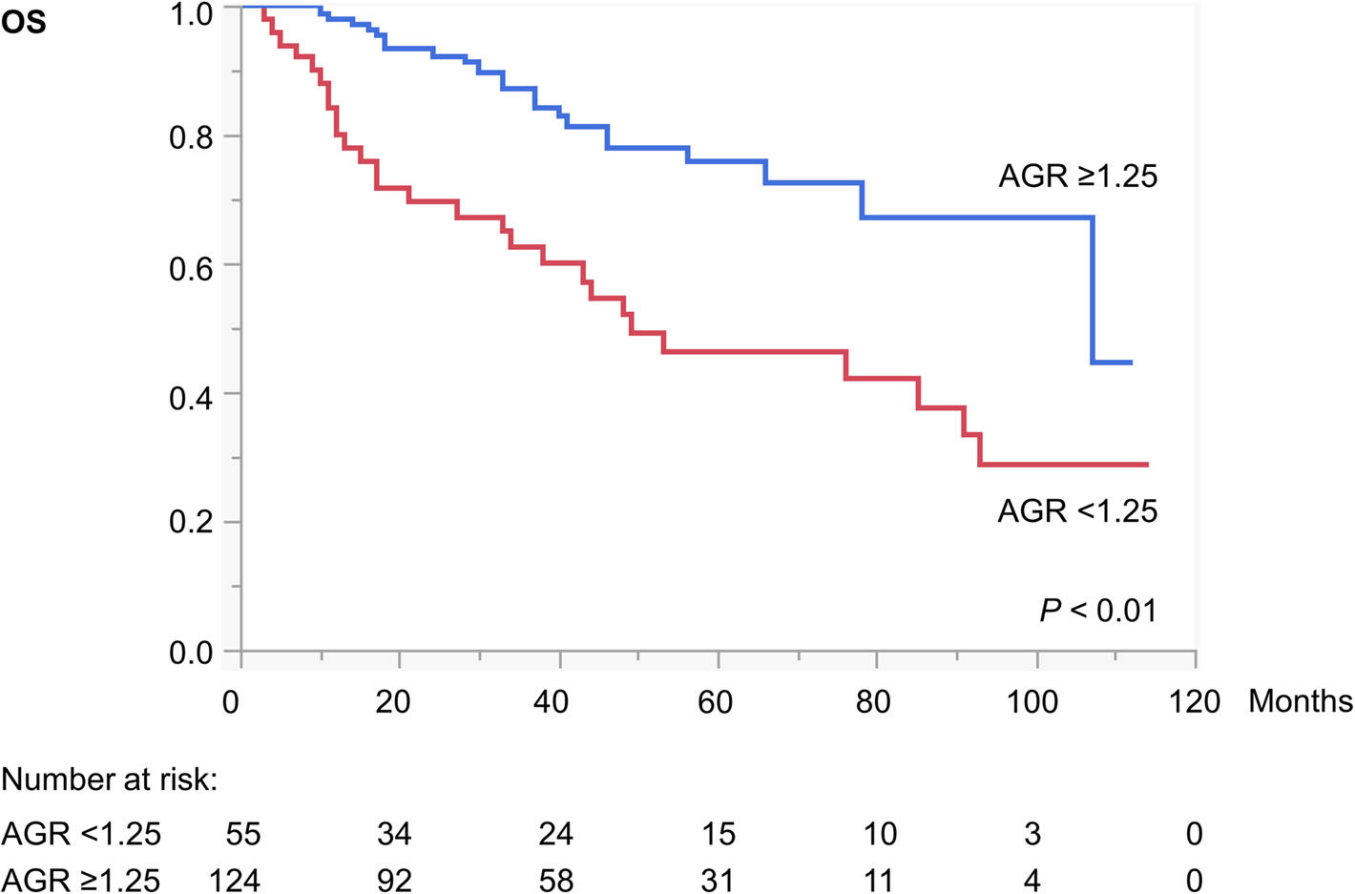
Kaplan–Meier curves depicting OS a in patients with AGR < 1.25 versus ≥1.25 (*P* < 0.01, log-rank test). Abbreviations: AGR = albumin-to-globulin ratio; OS = overall survival

Kaplan–Meier curves with log-rank tests showed significant associations of preoperative AGR < 1.25 with shorter CSS (Fig. 2) and OS (Fig. 3). Multivariate Cox proportional hazard regression analyses identified preoperative AGR < 1.25 as an independent poor prognostic factor for both CSS (hazard ratio [HR] = 2.81, *P* <  0.01) (Table 2) and OS (HR = 2.09, *P* <  0.05; Table 3).

**Table 2:** Univariate and multivariate Cox proportional hazard regression analyses of CSS

Parameter	Cutoff	Univariate		Multivariate	
		HR (95% CI)	*P*	HR (95% CI)	*P*
Age at surgery	Continuous	1.03 (0.99 to 1.07) per score	0.07	1.03 (0.99 to 1.08) per score	0.18
Sex	Male	Reference	< 0.05*	Reference	0.14
	Female	2.15 (1.09 to 4.13)		1.73 (0.83 to 3.51)	
Surgical technique	Open	Reference	0.16	Reference	0.21
	Laparoscopic	0.50 (0.23 to 1.34)		2.26 (0.63 to 8.93)	
Tumor location	Renal pelvis or both	Reference	0.56	Reference	0.72
	Ureter	1.21 (0.63 to 2.33)		1.14 (0.55 to 2.42)	
Tumor grade	G1/2	Reference	< 0.01*	Reference	< 0.05*
	G3	4.10 (2.07 to 8.66)		2.56 (1.07 to 6.50)	
Pathological T stage	≤T2	Reference	< 0.01*	Reference	0.06
	≥T3	3.67 (1.88 to 7.59)		2.51 (0.96 to 6.70)	
Pathological N stage	N0/x	Reference	< 0.01*	Reference	0.20
	N1–3	4.82 (2.20 to 9.77)		2.21 (0.65 to 7.00)	
Adjuvant chemotherapy	Yes	Reference	< 0.05*	Reference	0.51
	No	0.49 (0.25 to 0.97)		1.36 (0.55 to 3.37)	
Previous or synchronous bladder cancer	No	Reference	< 0.01*	Reference	< 0.01*
	Yes	3.61 (1.83 to 6.92)		3.49 (1.66 to 7.19)	
AGR	≥1.25	Reference	< 0.01*	Reference	< 0.01*
	< 1.25	3.90 (2.02 to 7.79)		2.81 (1.34 to 6.10)	

Abbreviations: *HR* hazard ratio, *CI* confidence interval, *AGR* albumin-to-globulin ratio

*Statistically significant

**Table 3:** Univariate and multivariate Cox proportional hazard regression analyses of OS

Parameter	Cutoff	Univariate		Multivariate	
		HR (95% CI)	*P*	HR (95% CI)	*P*
Age at surgery	Continuous	1.03 (1.00 to 1.07) per score	< 0.05*	1.03 (0.99 to 1.07) per score	0.07
Sex	Male	Reference	0.08	Reference	0.23
	Female	1.72 (0.93 to 3.07)		1.48 (0.77 to 2.76)	
Surgical technique	Open	Reference	< 0.05*	Reference	0.60
	Laparoscopic	0.44 (0.23 to 0.98)		1.33 (0.47 to 4.03)	
Tumor location	Renal pelvis or both	Reference	0.92	Reference	0.88
	Ureter	1.03 (0.58 to 1.80)		1.05 (0.57 to 1.94)	
Tumor grade	G1/2	Reference	< 0.01*	Reference	< 0.05*
	G3	2.61 (1.49 to 4.67)		2.15 (1.03 to 4.52)	
Pathological T stage	≤T2	Reference	< 0.01*	Reference	0.27
	≥T3	2.30 (1.32 to 4.08)		1.54 (0.71 to 3.35)	
Pathological N stage	N0/x	Reference	< 0.01*	Reference	0.09
	N1–3	4.32 (2.14 to 8.12)		2.56 (0.87 to 7.09)	
Adjuvant chemotherapy	Yes	Reference	0.33	Reference	0.23
	No	1.36 (0.73 to 2.43)		1.62 (0.74 to 3.61)	
Previous or synchronous bladder cancer	No	Reference	< 0.01*	Reference	< 0.05*
	Yes	2.43 (1.28 to 4.39)		1.99 (1.01 to 3.75)	
AGR	≥1.25	Reference	< 0.01*	Reference	< 0.05*
	< 1.25	2.90 (1.66 to 5.15)		2.09 (1.12 to 3.92)	

Abbreviations: *HR* hazard ratio, *CI* confidence interval, *AGR* albumin-to-globulin ratio

*Statistically significant

## Discussion

The present study demonstrates that a decreased (< 1.25) preoperative AGR was an independent indicator of poor prognosis for CSS and OS of patients with UTUC treated with radical nephroureterectomy.

Clinicopathological factors that serve as prognostic factors for UTUC [5–15] include sex [5], age [6], tumor size [7, 8], ureteral involvement [9], and body mass index [10], as well as laboratory markers [11–15] such as the neutrophil-to-lymphocyte ratio [11, 12], albumin [13], hemoglobin [14], and the prognostic nutritional index [15]. On the other hand, the AGR has been reported as a useful biomarker in various malignancies [16–26], including urological cancers [20–26]. However, the significance of the AGR as a prognostic marker for UTUC has not been fully investigated [23–26]. For example, a study of a Chinese cohort of 187 operable patients with UTUC [23] demonstrated that AGR < 1.45 is an independent risk factor for poorer CSS and OS. Another study of a Chinese cohort of 620 patients with UTUC treated with radical nephroureterectomy found that AGR < 1.45 is an independent predictor of adverse pathologic features, recurrence-free survival, CSS, and OS [24]. Similarly, analysis of a Japanese cohort of 124 patients with UTUC undergoing radical nephroureterectomy identified AGR < 1.40 as an independent prognostic factor for recurrence-free survival, CSS, and OS [25]. Finally, another Japanese study comprising 105 patients with UTUC undergoing radical nephroureterectomy reported that AGR < 1.24 was an independent predictor for both worse disease-free and overall survivals [26]. Our results are consistent with those of these previous reports and add further evidence in this field.

The association between a low AGR and poor outcome of patients with cancer requires further research. However, the available data show that poor nutritional status or hypoalbuminemia is a negative prognostic factor for certain malignancies [13, 15, 16]. Chronic inflammation involving serum globulins plays a crucial role in tumor proliferation, immune evasion, and metastasis. These serum globulins secreted by tumor-related cells reportedly promote tumor development, immunosuppression, and metastasis [16]. A low AGR may thus reflect the degree of poor nutritional status (hypoalbuminemia) and tumor progression (hyperglobulinemia) in a more sensitive manner than either measure alone and may therefore serve as a highly significant prognostic biomarker. Based on a similar concept as AGR (i.e. use of a ratio), several systemic inflammatory markers, such as the neutrophil-to-lymphocyte ratio [11, 12, 28], platelet-to-lymphocyte ratio [28], and lymphocyte-to-monocyte ratio [28], have been established and well-investigated in the field of oncology, including urothelial carcinoma.

The major limitations of this study are its retrospective, single-institutional design and the limited number of patients. Further studies with larger populations are needed to confirm our results.

## Conclusions

Given the significant prognostic associations of the AGR with CSS and OS, AGRs are easy to determine in routine clinical practice, and a preoperative AGR < 1.25 might serve as a useful prognostic biomarker of patients with UTUC treated with radical nephroureterectomy.

## Supplementary information

**Additional file 1: Table S1.** The REporting recommendations for tumour MARKer prognostic studies (REMARK) checklist of the present study (based on the original guideline [27]).

## Data Availability

Because of ethical restrictions, the raw data underlying this study are available from the corresponding author upon reasonable request.

## Abbreviations

AGR: Albumin-to-globulin ratio
AUC: Area under the curve
CSS: Cancer-specific survival
IQR: Interquartile range
OS: Overall survival
ROC: Receiver operating characteristic
UTUC: Upper tract urothelial carcinoma
